# Investigating the microwave heating behaviour of lunar soil simulant JSC-1A at different input powers

**DOI:** 10.1038/s41598-021-81691-w

**Published:** 2021-01-22

**Authors:** Sungwoo Lim, James Bowen, Giulia Degli-Alessandrini, Mahesh Anand, Aidan Cowley, Vibha Levin Prabhu

**Affiliations:** 1 School of Physical Sciences, The Open University, Milton Keynes, MK7 6AA UK; 2 School of Engineering and Innovation, The Open University, Milton Keynes, MK7 6AA UK; 3 European Astronaut Centre, Cologne, Germany; 4 Department of Earth Sciences, The Natural History Museum, London, SW7 5BD UK

**Keywords:** Mineralogy, Petrology, Mechanical properties, Civil engineering

## Abstract

For a sustainable human presence on the Moon, it is critical to develop technologies that could utilise the locally available resources (a.k.a. in situ resource utilisation or ISRU) for habitat construction. As the surface soil is one of the most widely available resources at the Moon, we have investigated the viability of microwave heating of a lunar soil simulant (JSC-1A). JSC-1A was thermally treated in a bespoke microwave apparatus using 2.45 GHz frequency, using five different microwave powers in the range of 250 W to 1000 W. The structural properties of the resulting products were analysed to determine whether their microstructures and mechanical strengths differ under different input powers; and whether input power plays a crucial role in triggering thermal runaway, for identifying the optimum power for developing a microwave-heating. Our key findings are: (i) the higher input powers (800 W and 1000 W) generate the highest yields and microstructures with the greatest mechanical strengths, at the shortest fabrication times, and (ii) thermal runaway improves the microwave heating efficiency despite the rapid increase in temperature, once it is triggered. Our findings are of key importance for developing a microwave-heating payload for future lunar ISRU demonstration missions, contributing towards 3D printing-based extra-terrestrial construction processes.

## Introduction

A new wave of space exploration and the ambition for a permanent presence of humans on other planetary bodies such as the Moon have necessitated research into the construction and resource utilisation in extra-terrestrial environments^1^. The current efforts are directed towards building lunar habitats and infrastructures, such as radiation protection shields, surface pavements, bridges, dust-shield walls and spacecraft landing pads using resources available in-situ. A key technology most likely to be employed in the lunar construction process(es) is a robotic 3D Printing platform^2^ because of its uncomplicated and autonomous operation.

Microwave heating is considered a more viable fabrication method for a 3D Printing platform compared with solar^3^ and laser^4^ sintering, as this technique does not depend on the availability of sunlight and only requires ~ 23% of the energy compared to that for laser sintering^5^ with reduced fabrication time, due to the volumetric heating that is intrinsic to the process^6^. Microwave heating causes sintering and melting of the feedstock. Sintering is achieved when particles are fused together at temperatures below the material’s melting point. The low thermal conductivity of lunar soil and simulants^7^ means that subsurface heating of the soil using a laser or solar energy would be challenging due to inefficient heat transfer by conduction. In contrast, microwave energy penetrates deeper into the lunar soil and is thus efficient in heating the subsurface^8^. Furthermore, the broad range of particle size distribution of lunar soil and simulant (0.002–5 mm^9^) contributes towards enhanced sintering effects. A preliminary study involving microwave heating experiments, using a domestic microwave oven with 1000 W of input power, demonstrated that 35 g of a lunar soil simulant (JSC-1A) sintered/melted in 480 s, making JSC-1A an ideal material for further testing^10^.

**Figure 1:**
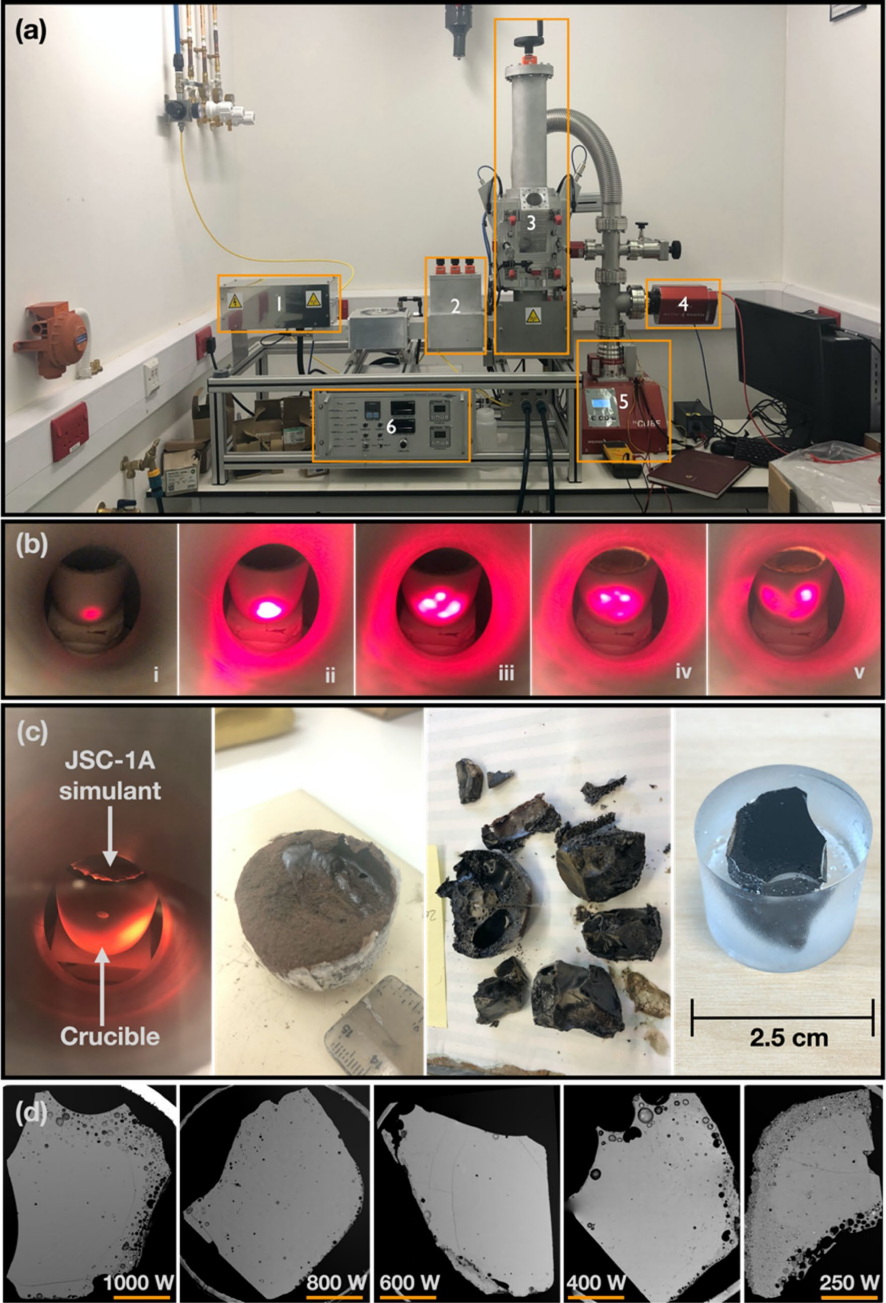
(**a**) The bespoke microwave heating equipment located at The Open University, used for microwave heating experiments on JSC-1A. The dimensions of this microwave apparatus are 860 mm (L) × 340 mm (W) × 1037 (H) mm, and it has been designed to allow a user to control the input power, withstand a high temperature of the specimen (> 1400 °C) while allowing accurate monitoring of changes in temperature, pressure and volatile output of the system during heating. The labelled parts are—1: magnetron, 2: three stub tuner, 3: vertical tuner and specimen chamber with a viewfinder, 4: mass spectrometer, 5: turbo (vacuum) pump, 6: controller and power supply unit. (**b**) A time-step series showing a crucible with JSC-1A being heated in the microwave. From left to right, it depicts a hotspot formation by thermal runaway for 30 s. When the temperature inside the specimen reaches the threshold (620–700 °C) of thermal runaway^14^, a hotspot occurs as a single dot and grows up to ~ 1 cm with maximum temperature over its melting point (> 1150 °C for JSC-1A) within a few seconds causing instant melting of the material at the hotspot position. The hotspot moves and splits, possibly due to the different microwave energy absorption rate caused by the inhomogeneous chemical composition of the specimen. (**c**) A snapshot of an ongoing microwave experiment on JSC-1A at 1000 W of input power. Images from left to right show how the specimen was sliced into a polished block for analysis. (**d**) The SEM BSE image overviews of the five samples analysed in this work. Note that all images have the same scale (5 mm) indicated by an orange bar in each case.

In this paper, we report on the outcome of a series of experiments on JSC-1A that was subjected to microwave heating at atmospheric pressure but at different input powers using a bespoke 2.45 GHz microwave equipment (Fig. 1a). The objectives are to investigate (i) how the microstructure and mechanical strengths of the sintered/molten specimens are affected by different input powers while keeping the total input energy at 900 kJ for each specimen, and (ii) whether input power is a major factor in order for a thermal runaway to occur during heating. Thermal runaway is defined as an unstable macroscopic phenomenon related to the dielectric properties of material during microwave heating. A dramatic temperature increase occurs while the applied microwave power remains constant^11^, causing the occurrence of a local “hotspot”^12^ (Fig. 1b). It is one of the most critical issues when processing material with temperature-dependent dielectric properties^13^, which is also applicable to the heating of lunar soil/simulants.

The findings from this work have been used to suggest the optimal input power for microwave heating of lunar soil, and the minimum input power for future lunar in situ Resource Utilisation (ISRU) activities, including the development of a microwave-heating payload, to identify fundamental criteria for a microwave heating-based resource extraction and 3D printing platform as part of an extra-terrestrial construction process.

## Results

A total of five different microwave input powers were applied to a starting mass of 50 g of JSC-1A which yielded hardened mass in each case. Note that *yield* here is defined as the mass of the hardened specimen after thermal processing with respect to the original mass of the untreated raw material. After the thermal processing, all specimens displayed three distinct microstructural areas (fully-molten, partially-molten and sintered) depending upon the extent of heating experienced in each case. The fully-molten areas are mostly glassy, with occasional relict mineral grains. The partially-molten areas are characterised by a predominance of vesicular glass with embedded mineral grains—some of which are relict grains while others are newly-formed from the melt as apparent through their texture. The sintered areas occur mostly at the outer edges of the specimens. They typically comprise a mixture of (i) un-melted mineral grains such as olivine and plagioclase with evidence of chemical interactions at the grain-glass interface, (ii) a glass matrix dotted with newly formed minerals, and (iii) numerous vesicles.

### 1000 W input power/yielded 100% (50 g/50 g)

**Figure 2:**
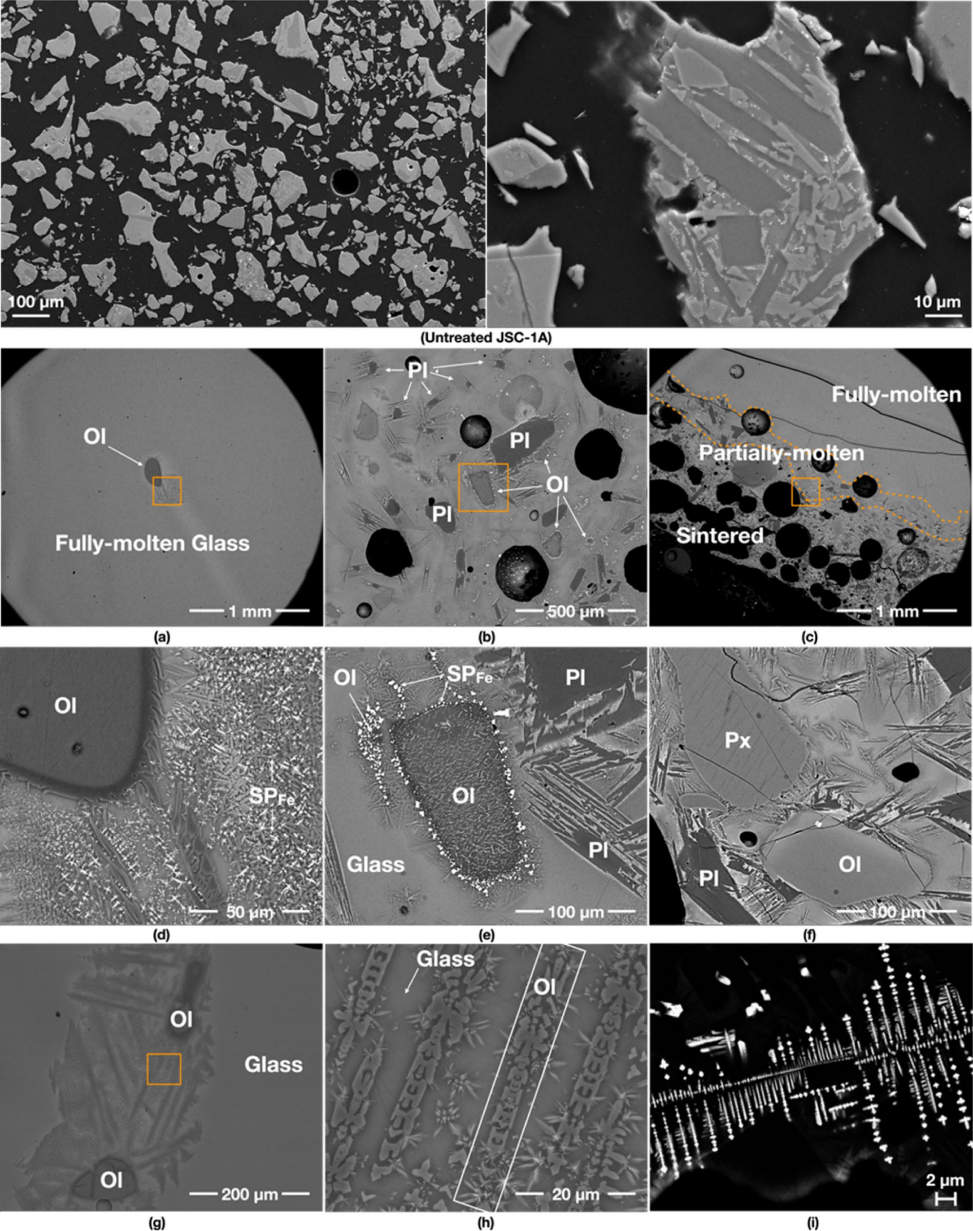
Back scatter electron (BSE) images of the 1000 W specimen. The first two images show untreated JSC-1A. (**a**–**c**) show an overview of its fully-molten, partially-molten and sintered areas, respectively. (**b**,**c**) contain an abundance of mineral grains—olivine (Ol), plagioclase (Pl) and Fe-rich spinels (SP_Fe_), embedded in a glassy matrix, and large vesicles. (**d**) is the detail of the box in (**a**) showing an oblate olivine grain (Ol) with a darker reaction rim caused by chemical re-equilibration with the melt. (**e**) is the detail of the box in (**b**) indicating SP_Fe_ formation from an olivine grain. (**f**) is the detail of the box in (**c**), showing the partial reaction of pyroxene (Px), olivine (Ol) and plagioclase (Pl) with the melt. (**h**) (the detail of the box in (**g**)) depicts the atypical dissolution shapes of olivine (Ol) with Dove- and H-shaped dendritic morphologies, which are highly euhedral with well-defined habit and growth pattern. Note that these patterns probably nucleated from the glass matrix. (**i**) shows iron oxide particles (likely to be titanomagnetite) arranged in orthogonal multiple cross-arm patterns growing euhedrally in a sintered area. Note that the contrast-brightness was adjusted to optimise the clarity of the image.

The 1000 W specimen shows mostly fully-molten and partially-molten areas, as the supplied energy rate is much higher than that of other specimens despite the same total energy supplied in each case. The fully-molten areas are comprised of a homogeneous glass, without many vesicles and mineral grains (Fig. 2a). The glass in the proximity of the olivine grain is rich in newly formed dendritic minerals, indicating neo-crystallisation of olivine and Fe-rich spinels (SP_Fe_) from the melt (Fig. 2d). These are typical devitrification assemblages previously reported from natural basaltic glasses^15–17^.

The partially-molten areas are comprised of a glassy matrix containing relict plagioclase and olivine grains (Fig. 2b). Large plagioclase laths from the original JSC-1A powder have retained their original shapes while smaller grains seem to have partially dissolved into the glassy melt (Fig. 2d). A typical relict-grain of olivine has an oval shape, with darker overgrowths (i.e. more MgO-rich) as reaction rims and a dendritic pattern. A typical feature is the association of olivine with SP_Fe_ particles that preferentially seem to occur along the weaker zones in the olivine grains (Fig. 2e). In addition, some olivine grains in the partially-molten areas show unique recrystallisation textures with Dove- and H-shaped dendritic morphologies^16,18^. These form in the glass that surrounds large relict olivine grains (Fig. 2h). These morphologies are typical nucleation products that form during rapid cooling of a melt, without any associated SP_Fe_ particles (Fig. 2g).

Sintered areas are only limited to the edges of the specimen, showing an abundance of minerals; mainly plagioclase and olivine with few pyroxene grains (Fig. 2c,f), as well as unusual SP_Fe_ particles (Fig. 2i) arranged in orthogonal multiple cross-arm patterns growing euhedrally^19^. These minerals have jagged rims and needle-like/dendritic extensions, indicating chemical reactions with the surrounding melt/glass, resulting in neo-crystallisation of minerals.

### 800 W input power/yielded 90% (45 g/50 g)

**Figure 3:**
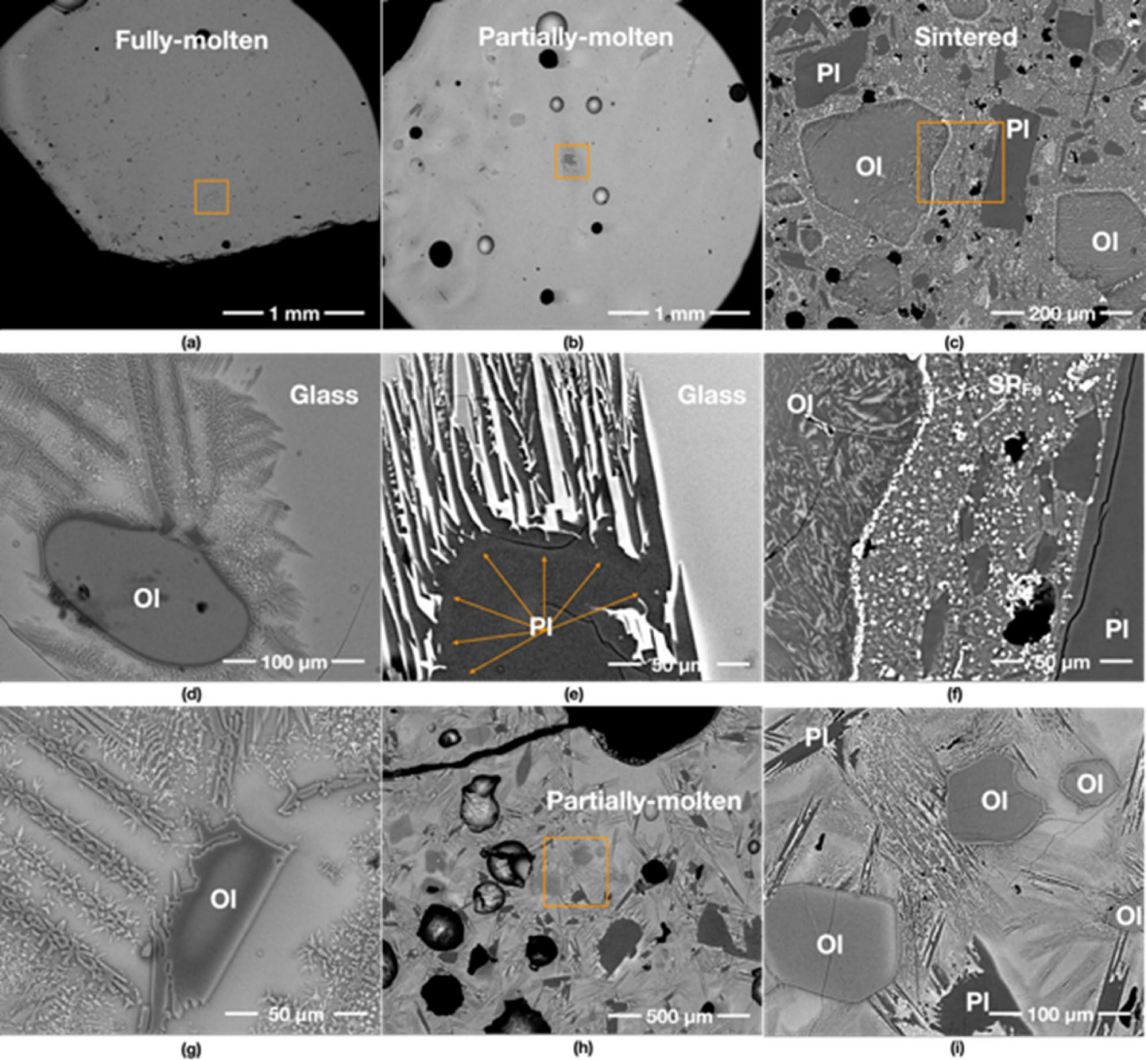
BSE images of the 800 W specimen. (**a**–**c**) show an overview of its fully-molten, partially-molten and sintered areas, respectively. (**e**) is the detail of the box in (**b**) showing the crystallisation of a plagioclase (Pl) grain with acicular projections, which is new growth upon crystallisation in the surrounding glass. As the grain did not fully melt, the core of the original grain is still preserved. (**f**) is the detail of the box in (**c**) depicting formed Fe-rich spinel (SP_Fe_) particles from an olivine grain and the glass matrix. (**d**,**g**) show the neo-crystallisation of olivine from the melt. Unlike (**d**), the olivine in (**g**) has a second light-coloured rim with a very angular shape indicative of new crystallisation during cooling. (**i**) is the detail of the box in (**h**), indicating that minerals are starting to melt in the partially molten area.

The fully-molten area of the 800 W specimen comprises mostly of a feature-free glass (Fig. 3a,d). The partially-molten area contains a few large vesicles around 120–250 μm in diameter (Fig. 3b) and some relict grains of plagioclase (Fig. 3e) and olivine. Olivine shows evidence of partial melting (Fig. 3h,i). The round morphologies of the olivine grains in Fig. 3d,g indicate resorption, followed by crystallisation in the melt of new olivine with the same Dove- and H-shaped dendritic morphologies which are seen in the 1000 W specimen. The olivine grains are also surrounded by feather-like dendritic minerals and tiny (less than 10 μm) SP_Fe_ particles. This type of olivine is found in all specimens. The plagioclase grain in Fig. 3e shows a rounded-shaped darker core with needle-shaped plagioclase extensions, indicating the grain has partially melted and neo-crystallised upon cooling.

The sintered areas exhibit different characteristics from 1000 W, likely as a result of the lower temperature experienced. The sintered areas contain abundant SP_Fe_ particles, scattered in the glass matrix and surrounding olivine grains (Fig. 3c). The fragments of the original JSC-1A minerals are still visible: plagioclase does not show any growth of needles, and olivine still preserves sub-angular edges. Furthermore, the interior of the olivine grains in the partially-molten areas is mostly featureless (Fig. 3h–i), while the interior of the olivine in the sintered areas is dotted by exsolving SP_Fe_ particles (Fig. 3f).

### 600 W input power/yielded 82% (41 g/50 g)

**Figure 4:**
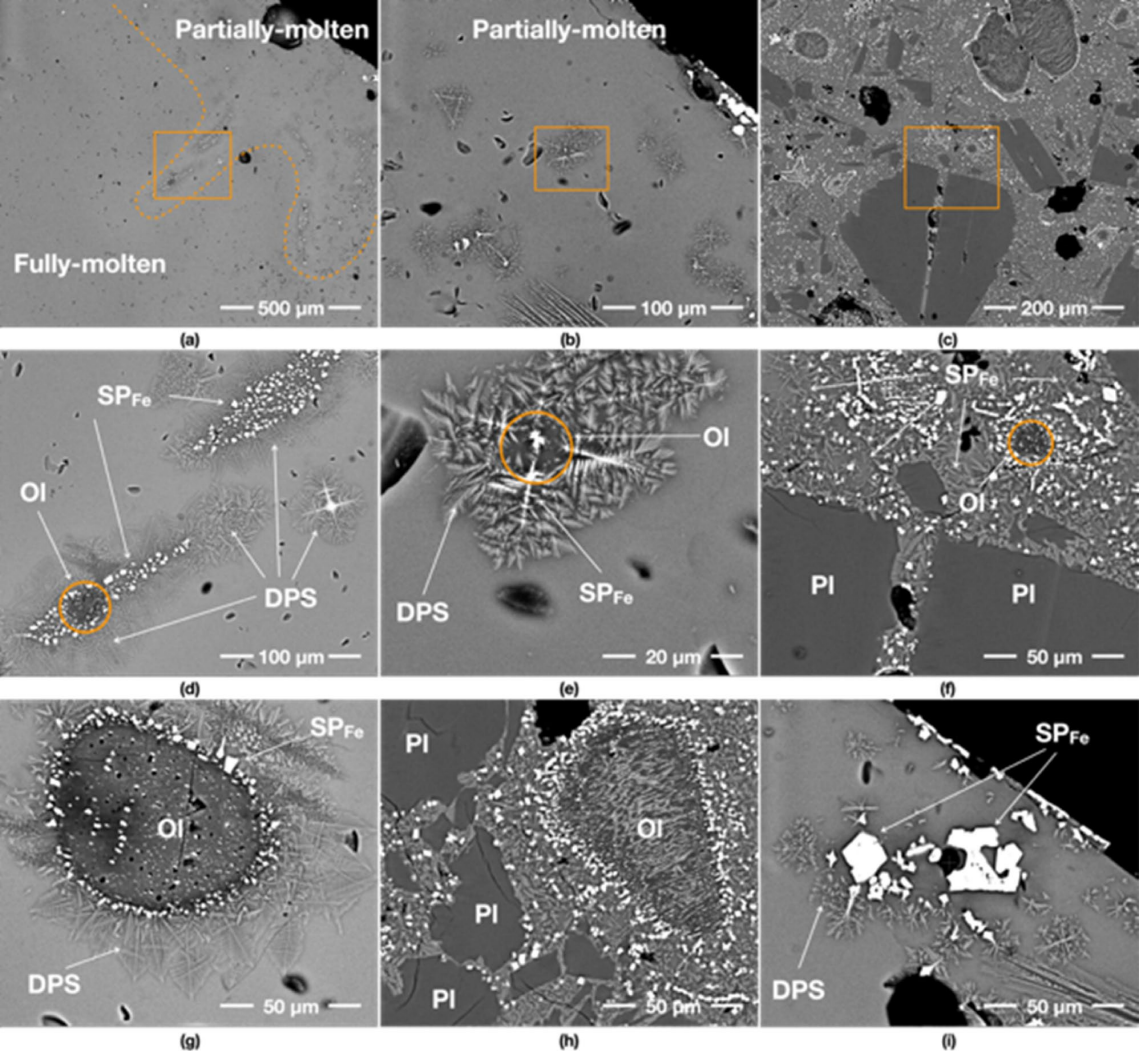
BSE images of 600 W specimen. (**a**–**c**) show an overview of its fully-molten, partially-molten, and sintered areas. (**d**) is the detail of the box in (**a**) indicating the olivine (Ol) is reacting with glass melt, and then formed dendritic patterned silicates (DPS) and rectangular-shaped SP_Fe_ particles. (**e**) is the detail of the box in (**b**) presenting another example of the olivine (Ol) reaction forming SP_Fe_ particles. (**f**) is the detail of the box in (**c**) showing abundant DPS and rectangular-shaped SP_Fe_ particles in the glassy matrix. (**g**) from a partially molten area. (**h**) from a sintered area shows that SP_Fe_ particles are exsolved from olivine grains with distinctive inner patterns, indicating that both grains may have undergone the same chemical processes with different peak temperatures and cooling rates. (**i**) shows unusually large SP_Fe_ particles formed in a partial-molten glassy matrix.

The fully- and partially-molten areas of the 600 W specimen contains more relict mineral grains than 1000 and 800 W specimens (Fig. 4a,b). Here, the olivine grains are reacting with silicate melt, to form feather-like dendritic minerals, which are compositionally closer to amphibole group of minerals, with rectangular shapes (Fig. 4d) or orthogonal multiple cross-arm patterns (Fig. 4e) of SP_Fe_. Such textures and mineral assemblages are commonly observed in the crystallisation products of a basaltic melt^15,16,20^.

The sintered areas of the 600 W specimen are much wider than 1000 W and 800 W specimens and contain many more vesicles with still visible original fragments of JSC-1A minerals. The specimen is also characterised by the pervasive nucleation of SP_Fe_ particles in the glassy matrix (Fig. 4c,f), around olivine rims (Fig. 4g,h), and in the centre of dendritic patterned silicates (Fig. 4i). Plagioclase grains are still subhedral in shape, with no growth of needles, and olivine still preserves sub-angular edges, similar to the 800 W specimen.

### 400 W input power/yielded 70% (35 g/50 g)

**Figure 5:**
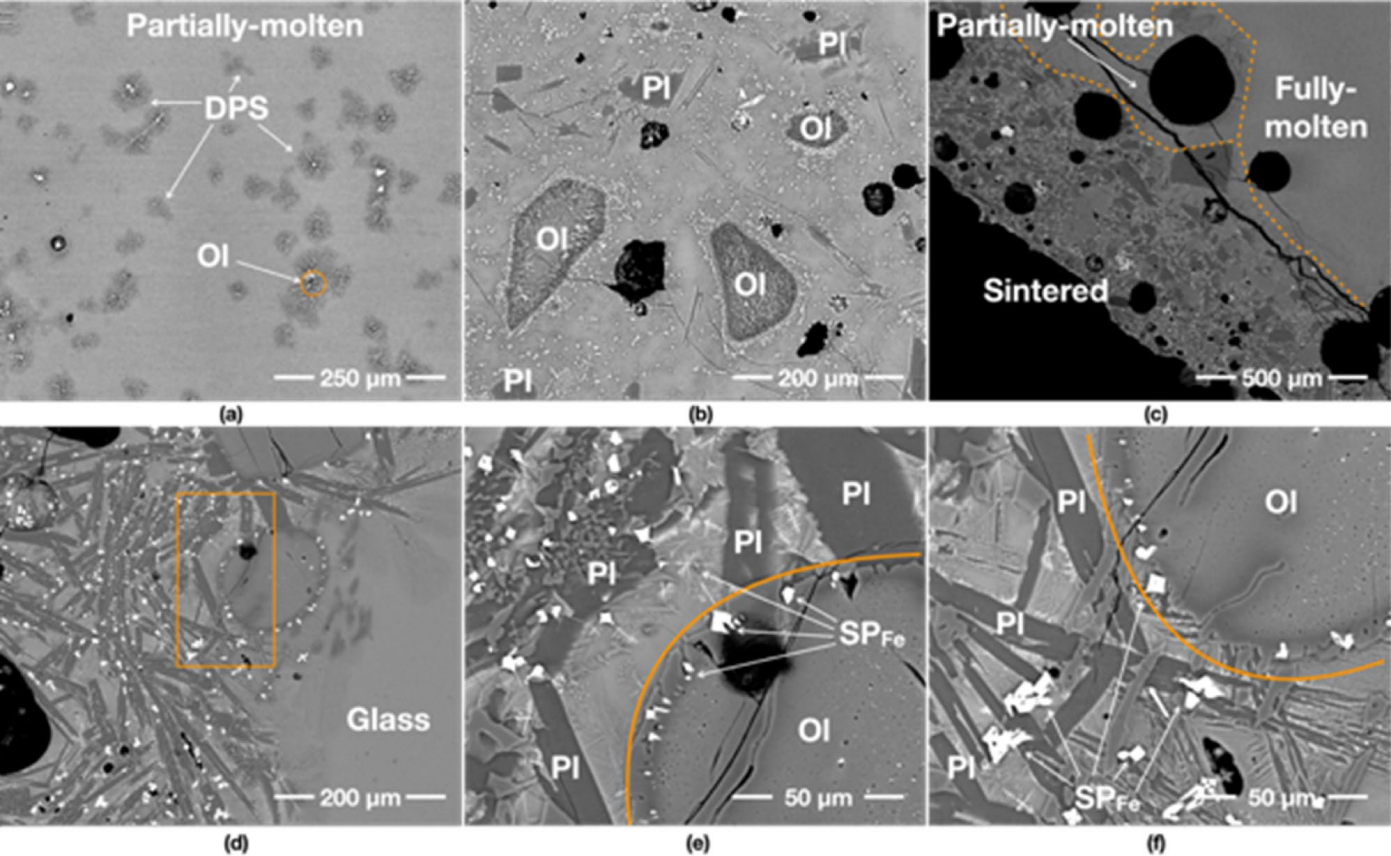
BSE images of 400 W specimen. (**a**–**c**) are an overview of its fully-molten, partially-molten and sintered areas. (**a**) Depicts many dendritic patterned silicate (DPS) formations in the partially-molten area. (**e**,**f**) are the details of the box in (**d**) showing large dendritic plagioclase (Pl) crystal growth and the formation of SP_Fe_ particles from an olivine (Ol) grain. (**e**,**f**) also show the trace of SP_Fe_ particle exsolution from the dark rim of an olivine (Ol) grain (see the dark rim near the orange curves).

The fully-molten areas of the 400 W specimen are comprised of uniformly distributed vesicle-like features containing abundant dendritic patterned silicates possibly due to lower heating temperature and/or longer heating times (compared to the higher power specimens (Fig. 5a). Mineral dissolution in the glassy matrix and occasional SP_Fe_ particle exsolutions from olivine are also observed. Most of the characteristics of the partially-molten and sintered areas in the 400 W specimen are similar to other specimens (Fig. 5b,c); however, from the visual inspection, the amount of vesicles is considerably lower than the 800 W and 600 W specimens. Nucleation of SP_Fe_ particles is still abundant in the partially-molten and sintered areas.

Figure 5d–f show large dendritic plagioclase crystal growths, which are found in a small area of 400 W specimen only. This type of dendrite feature indicates that the area might have experienced a higher cooling rate, e.g. up to 1000 °C/h as was reported by Lesher et al.^17^, than other areas.

### 250 W input power/yielded 44% (22 g/50 g)

**Figure 6:**
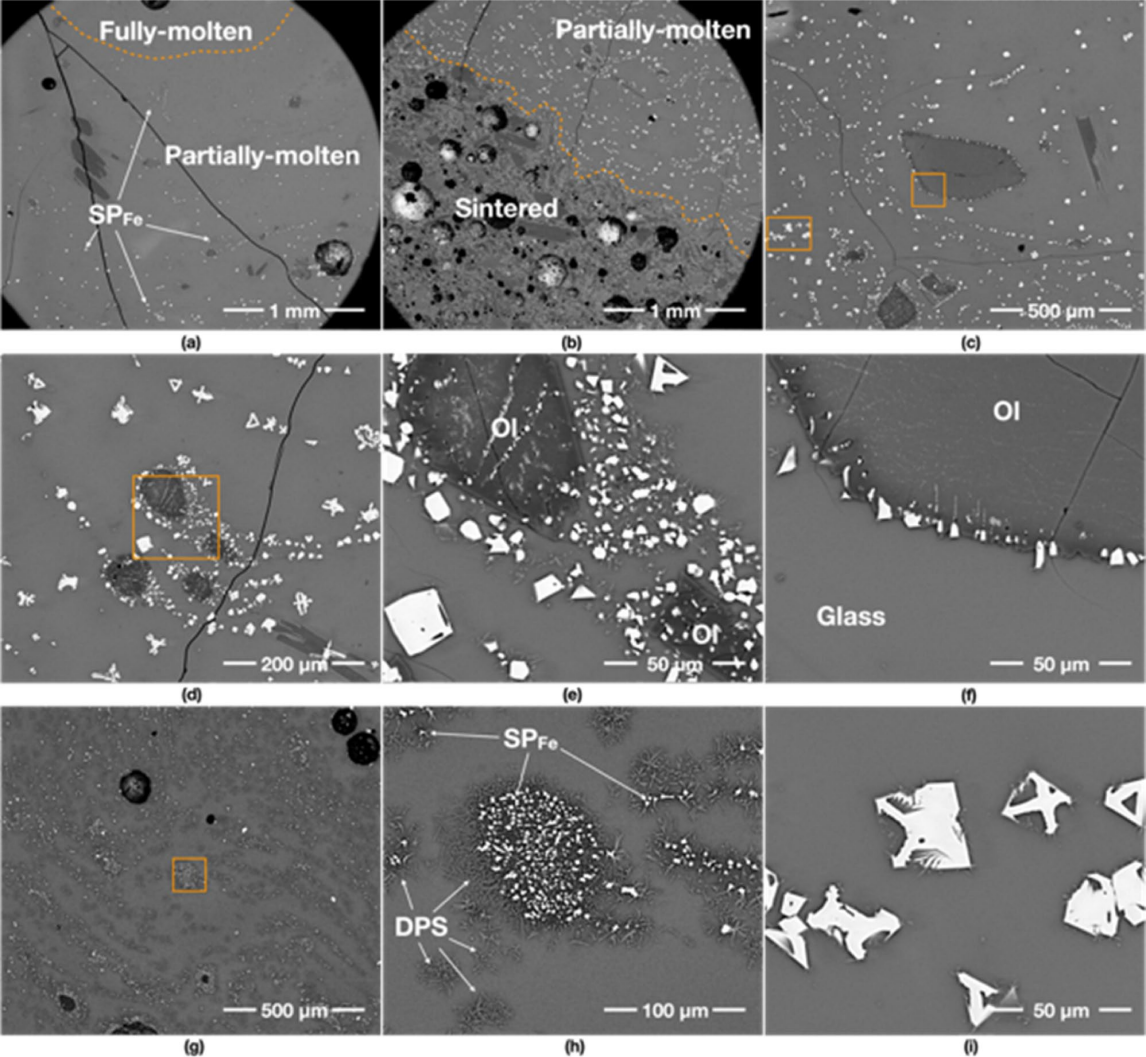
BSE images of 250 W specimen. (**a**,**b**) show an overview of its fully-molten, partially-molten and sintered areas, respectively. (**a**) indicates that fully-molten areas rarely exist and split among the partially-molten areas. The partially-molten areas in (**a**,**b**) have abundant SP_Fe_ particles with much fewer dendritic patterned silicates (DPS) than 400 W specimen over the entire area. (**e**) is the detail of the box in (**d**) showing the formed SP_Fe_ particles from an olivine (Ol) grain and the surrounding glass. (**h**) is the detail of the box in (**g**) representing the SP_Fe_ particles formed at the centre of DPS, which are possibly created through the neo-crystallisation of glass reacting with olivine (Ol) grains. (**f**) is the detail of the middlebox in (**c**) indicating the exsolving trace of SP_Fe_ particles from olivine (Ol) grain. (**i**) is the detail of the left box in (**c**) depicting the skeletal growth morphologies of SP_Fe_ classified as a cruciform type.

The 250 W specimen has very distinctive features, probably caused by longer heating time at relatively lower temperatures. Unlike other specimens, the 250 W specimen rarely exhibits fully molten areas. The partially-molten areas contain plentiful SP_Fe_ particles that are arranged in a circular/arc pattern (Fig. 6a,b), which is not seen in the specimens produced at higher input powers. This feature is normally observed in Si-rich andesite, where abundant spinel particles are formed in a glassy matrix under prolonged heating time (10 °C/min to 1300 °C)^21^. In a previous study, involving numerical simulation, it was found that the hotspot glow in the 200 W specimen experiences continuous high-temperatures over 1100 °C (except for the peak temperature of 1500 °C for a few seconds due to thermal runaway)^14^. Thus, we extrapolate that the core temperature of the 250 W specimen in the present experiment was around 1200 °C, which exceeds the melting point of JSC-1A (> 1100–1125 °C^22^). In the partially-molten area, SP_Fe_ particles are formed with a much larger size than other specimens, as shown in Fig. 6c–i. The trace of SP_Fe_ particle exsolution from an olivine grain is clearly shown in Fig. 6f as the SP_Fe_ particles are aligned with the darker rim of the olivine grain and have thin tails toward the inside of the olivine grain.

Furthermore, some SP_Fe_ particles of the 250 W specimen showcase complex geometric patterns (Fig. 6i). These particles are identified as the skeletal growth morphologies of titanomagnetite classified as a cruciform type^19^. They are also lager crystals than those observed at higher powers.

### Specimen temperature during microwave heating

**Figure 7:**
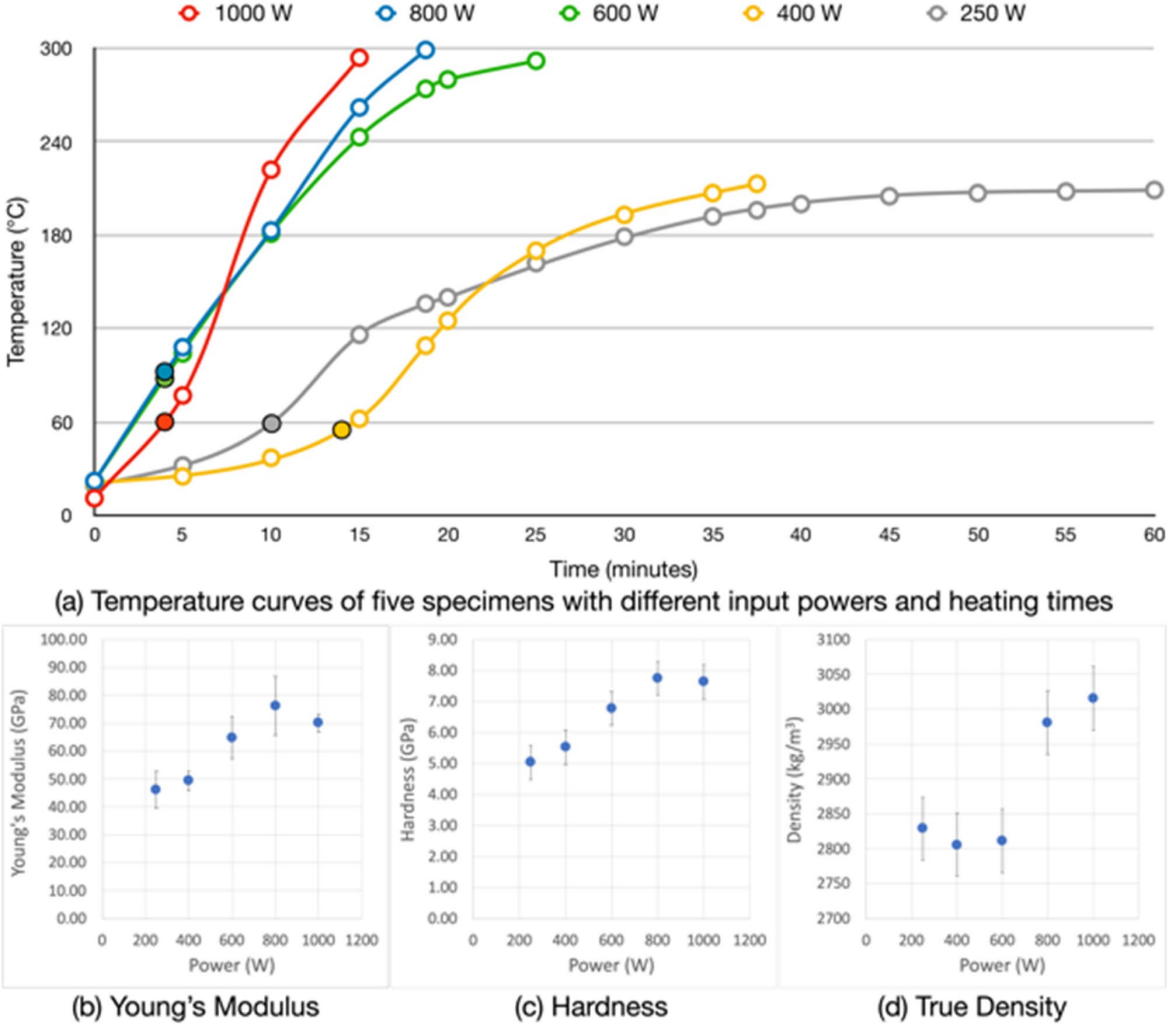
Temperature curves (**a**), Young’s modulus (**b**), hardness (**c**) and true density (**d**) of different input power specimen. Note that the temperatures in (**a**) correspond to the outer surface temperature of the crucible. The solid dots indicate the time when the hotspot caused by thermal runaway appeared on the crucible surface in 4 min for 1000 W, 3 min for 800 W and 600 W, 14 min for 400 W, and 10 min for 250 W specimen. The size and colour of the hotspot glow appear to reduce gradually from 1000 to 250 W. For example, the hotspot glow of the 1000 W sample was a bright yellow colour and > 2 cm in diameter, while that of the 250 W sample was a dimmed red colour with less than 1 cm diameter. The estimated temperature of the hotspot glows are between 1700 and 2200 °C depending on the sample mass and input power based on the previous numerical simulation study^14^.

Figure 7a shows the temperature curves of the five specimens that were heated under different input powers with the corresponding duration of heating, for a total energy dose of 900 kJ. Note that in this experimental setup, the recorded temperatures are for the outer crucible surface instead of the specimen surface (see “Methods” section), and the hotspot of each specimen occurred outside the pyrometer focus. Moreover, the specimens were surrounded by a ceramic paper to prevent thermal shock to the alumina crucible, which results in significant thermal insulation. Thus, the recorded temperatures shown in Fig. 7a are much lower than what the specimens experienced. With a separate experiment directly measuring the hotspot position on the crucible surface, the temperature was over 1200 °C, which is reasonable as the temperature of the hotspot caused by thermal runaway is around 1700–2200 °C^14^ considering the heat absorption of the crucible and ceramic paper.

As expected, the graph shows that higher input powers reached higher peak temperatures in a shorter time, whereas the peak temperatures for lower input powers were reached more slowly. All five specimens experienced thermal runaway with bright hotspots on the crucible surface (see the solid symbols in Fig. 7a). Regardless of the absolute temperatures in each case, it is interesting to note that the temperature curves of the five specimens represent a consistent heating trend, including thermal runaway, which results in a radical temperature increase.

Figure 7a also shows a flattening of the temperature curves towards the end of their heating cycles, possibly caused by a higher radiative heat loss than heat absorption. This is notably affecting the 400 W and 250 W specimens and may indicate a lower efficiency of microwave heating below 600 W of input power, caused by much longer heating times.

### Thermal runaway and the temperature curves

Unlike the observation of Liu and Sheen^11^, who concluded that thermal runaway has an input power threshold, it was observed in our previous simulation^14^ that thermal runaway occurs when the temperature of the lunar soil exceeds 620–700 °C, regardless of the input power. This temperature is close to the glass transition temperature of the JSC-1A powder (665–695 °C^23,24^). The experimental results presented here found that even the 400 W and 250 W specimens experienced thermal runaway despite their lower peak temperatures and slower temperature increase. This corroborates the simulation results and suggests that temperature is the key threshold for thermal runaway, and not the input power (although higher input power allows an individual specimen to experience thermal runaway sooner than those at lower input powers).

Despite being heated with the highest input power, the 1000 W specimen experienced thermal runaway around one minute after that of 800 W and 600 W specimens. This suggests that the 1000 W specimen experienced a slightly shorter period of a thermal runaway than expected, which may affect its hardness, resulting in the similar mechanical properties to the 800 W specimen. The rate of power absorption varies with the specimen’s volume and dimensions, hotspot position, and material’s particle size distribution and mineralogy. The first three elements can be controlled; while the particle size distribution and the mineralogical inhomogeneities of the specimen cannot. Although the variations described here apply to all specimens, these factors may have contributed to the 1000 W specimen more than other specimens.

### Physical and mechanical properties

The mean Young’s Modulus (70.0, 76.3, 64.8, 49.3, 46.2 GPa) and the hardness (7.73, 7.75, 6.78, 5.52, 5.04 GPa) of each specimen (1000 W to 250 W) show that there is a positive correlation between the input powers and Young’s Modulus and Hardness (Fig. 7b,c). Higher input power allows for a higher heating rate, effectively resulting in a homogeneous melting with fewer defects; thus, achieving higher hardness. This is true for all input powers except for the 1000 W specimen, which has values similar to the 800 W specimen. One of the possible reasons for this is that the properties may not change above a specific input power, i.e., 800 W could be the threshold of input power for the maximum hardness. This needs to be verified by another experiment with higher than 1000 W input power.

The true density of JSC-1A powder is 2904 kg/m^3^. The true density analysis of the microwave heated samples indicates that the 1000 W input power produces highest average density, i.e., 3.02 g/cm^3^, compared to 2.98 g/cm^3^ for 800 W, 2.81 g/cm^3^ for 600 W, 2.81 g/cm^3^ for 400 W, and 2.83 g/cm^3^ for 250 W (Fig. 7d). Note that the hardness measurement applies to the material surface only, so the density may not have any explicit correlation with hardness. In general, the density of hardened concrete can be categorised as: lightweight (1.44–1.84 g/cm^3^), normal (2.24–2.40 g/cm^3^) and heavyweight (> 2.60 g/cm^3^). As expected for mafic rocks, all specimens have much higher densities than the heavyweight concrete, regardless of input power. Le et al.^25^ showed that the density and compressive strength of well-printed concrete could exceed casted concrete. Thus, it is safe to consider that the compressive strength of 3D printed lunar soil/simulants may be stronger than heavyweight concrete as long as it is printed well with minimal gaps between the printed filaments.

## Discussion

The SEM analysis of the five specimens indicates that input power plays a vital role in differentiating the overall microstructure and mineral makeup of each specimen. Some minerals, including olivine and plagioclase feldspar, show distinctive partial melting and neo-crystallisation patterns. The temperature of a hotspot caused by thermal runaway can easily exceed the melting point of the mineral assemblage such that all specimens have a fully-molten area in their core and partially-molten and sintered areas further away from the core.

Fully-molten and partially-molten areas are mostly glassy with a few relict grains. In the fully-molten areas, 1000 W of input power produces the most homogeneous microstructure, i.e. mostly glass, devoid of any vesicles and minerals. The fully-molten areas indicate that any volatiles originally present either escaped very efficiently owing to the low viscosity of the melt or perhaps they are still dissolved in the melt and retained as a result of rapid quenching. On the other hand, 800 W and 600 W of input powers produced slightly inhomogeneous microstructures compared to that of 1000 W, with abundant vesicles and partially dissolved/reacted minerals. One possible reason for the occurrence of abundant vesicles is that because the system has more partial melting with higher viscosity due to lower maximum temperature than that of 1000 W, it makes less easy for the released volatiles to escape from the heated material. However, both 400 W and 250 W of input power produced again vesicle-free molten areas, presumably due to much-prolonged heating time to help volatiles escape or dissolve into the matrix, with pervasive neo-crystallisation of square-shaped SP_Fe_ and DPS. Considering a 3D printing application, 1000 W (50 g, 100% yield) and 800 W (45 g, 90% yield) microwave energy for a 50 g of specimen show the best outcomes in terms of yields, homogeneity of the molten material and strength. As the experiment was set to the same total input energy of 900 kJ, these specimens prove that higher input powers are more efficient for savings in fabrication time and energy.

All specimens produced abundant SP_Fe_ particles in both sintered and partially molten areas regardless of the different input powers, although 1000 W and 800 W specimens have considerably less of them. These SP_Fe_ particles are neither magnetite (Fe_3_O_4_) nor haematite (Fe_2_O_3_) end members. Some of them were exsolved from large olivine grains, which are also reported in^26^ for another lunar simulant JSC-2A, while others were formed directly from the glass matrix. Interestingly, the partially molten area of the 250 W specimen forms plenty of SP_Fe_ particles arranged in broadly circular/arc patterns, which is not seen in the specimens formed at higher input powers. The patterns are too large to be tracing the boundary of former mineral grains; thus, some other explanation is needed. We hypothesise that these patterns could have formed as a result of either (i) convection currents in the molten material, or (ii) perlites, which are curved/concentric cooling structures created upon cooling of the glass. Numerous dendritic patterned silicates of 400 W and SP_Fe_ particles of 250 W specimens in the partially molten area indicates possible nucleation from the melting olivine or nucleation from the glass matrix. Because this feature is lacking in the higher input power specimens, the prolonged heating times of both 400 and 250 W specimen (up to four times longer compared to 1000 W) could be the cause of the abundant dendritic silicates and SP_Fe_ formation despite the relatively lower peak temperatures. Regardless of the exact mechanism(s) of formation, the abundance of SP_Fe_ particles in low-power specimens suggests that it might be easier to extract iron from partially molten and sintered soil rather than untreated soil because SP_Fe_ particles are effectively separated from minerals containing Fe mixed with other elements, such as Si and Mg comprising silicate minerals. Although the mechanism of iron extraction is beyond the scope of this work, the observation suggests that microwave heating seems capable to facilitate the first step to segregate iron out of silicates. Thus, it could be considered as a possible method to enrich the lunar soil feedstock for iron extraction combined with various methods (e.g. isothermal reduction using a reducing agent—H_2_ or CO^27^).

The microstructures of the sintered areas, excluding the 1000 W specimen, all look similar due to the relatively lower temperatures experienced by the outer portions of the specimens. Grains are angular/subangular, and there is not acicular/needle-like growth of plagioclase. Glass matrix in between grains has melted and subsequently crystallised as a micro-crystalline matrix of SP_Fe_ particles and plagioclase. Nevertheless, the original powder morphologies can still be identified. SP_Fe_ particles are widespread in the matrix and in and around olivine grains. However, for 3D printing applications using lunar soil, sintered (instead of fully- or partially-molten) material suffers from a few drawbacks: (i) it reduces solid yields, resulting in lower energy efficiency; (ii) it produces an inhomogeneous microstructure with abundant vesicles, causing low strengths overall; and (iii) unmelted material may increase friction and material segregation causing unwanted lumps inside the delivery path to the printing nozzle for an extrusion-based 3D printing technique, which is the most appropriate technique to fabricate large-scale construction components on-site. For these reasons, higher input powers would be preferable for a microwave heating-based 3D printing technique, particularly for an extrusion-based method.

Finally, the most notable findings from this work are summarised as below.

- Higher input powers generate more homogenous specimens, with higher yields in less fabrication time. This means the fabrication of these specimen is more energy-efficient than for lower input powers. They also generally display larger proportions of fully-molten areas to sintered areas and are mechanically stronger and denser than lower-power specimens.
- Different heating rates associated with each input power can be utilised for different purposes. For 3D printing, higher input powers, i.e. 1000 W or 800 W in the case of our experiment, per 50 g of lunar soil might be an optimal input power in terms of energy efficiency and printing time. On the other hand, lower input power with longer heating times may serve other purposes such as segregation of SP_Fe_ particles—although more experiments under vacuum condition should be conducted to validate this possibility further.
- 250 W of input power results in a specimen with low yields, lower fractions of fully molten material, lower peak heating temperatures and considerably longer fabricating times (up to an hour vs 15 min at 1000 W). Given the properties of the 250 W specimen, 250 W should be considered as a minimum power for sintering/melting lunar soil and simulant JSC-1A. Less than 250 W make the process unreasonably inefficient due to the radiative heat loss caused by longer heating times. This finding confirms previous simulations^14^ and lab experiments^10^.
- Thermal runaway is a phenomenon that generally needs to be avoided in industrial applications, due to uncontrollable and rapid increase in temperature. In our case, however, thermal runaway seems particularly favourable as it allows instant melting at a ‘hotspot’ location. Thermal runaway has a temperature threshold (620–700 °C) even with the lowest input power 250 W, and it is related to the transition phase and the dielectric properties of the material. As the hotspot location can be expanded, split or even moved by temperature threshold (Fig. 1b), it may be possible to control where the temperature is raised, and thus target where thermal runaway will occur^14^. Control on the position of a thermal runaway could be exploited in microwave heating-based 3D printing for lunar habitat construction and resource extraction, to maximise melting efficiency.
- Microwave heating of lunar soil could facilitate the first step to segregate iron out of silicates, to be then extracted by various methods (e.g. isothermal reduction using a reducing agent—H_2_ or CO) for other ISRU applications.

## Methods

### Experiment material: lunar soil simulant JSC-1A

The material used for this experiment is a lunar soil simulant, JSC-1A, compositionally broadly similar to low-Ti mare soil, developed by NASA’s Johnson Space Centre (JSC). JSC-1A is a reproduction of the original JSC-1 simulant: a crushed volcanic tuff with abundant glass and a large amount of nano-meter sized magnetite (Fe^2+^Fe^3+^_2_O_4_) particles^28^. JSC-1 simulant was originally collected from the southern vents of Merriam Crater in San Francisco volcanic field, north of Flagstaff, Arizona, USA^29^. The volcanic tuffs were used to mimic the particle size distribution and engineering properties of Apollo 14 lunar soil^30^, while the volcanic sediments were used to simulate its chemical composition^31–33^. Although JSC-1 and JSC-1A simulants closely resemble the real lunar soil in many aspects, they cannot replicate some unique lunar features. For example, the nano-phase iron (np-Fe^0^) globules found on the rims of agglutinitic glass and vapour-deposited glass caused by space weathering, are a major challenge to replicate in soil simulants^34^.

Table 1 summarises the bulk chemical compositions of JSC-1, JSC-1A (from different references), Apollo 14 and Apollo 17 lunar soil samples. The bulk chemical composition of JSC-1A closely matches that of the actual lunar soil collected by the Apollo 17 mission^35^. The largest discrepancy is in the titanium content, with 1–2 wt% in JSC-1A simulant compared to ~ 5 wt% in the Apollo 17 sample. Although the total Fe contents of JSC-1A simulant and lunar soil are comparable, the simulant contains both Fe^2+^ (∼ 76%) and Fe^3+^ (∼ 24%) ions, as opposed to the lunar soil which contains only Fe^2+^ ions. Lack of Fe^3+^ in lunar soil is due to the prevalence of reducing conditions at the Moon, which means that iron in its soil may only exist as metallic Fe^0^ or as Fe^2+^ (in iron oxides or silicates)^30^.

**Table 1:** Chemical composition, expressed as weight%, of lunar soil and simulants.

Major element composition	Percentage by weight (wt %)					
	JSC-1^33^	JSC-1A^38^	JSC-1A^33^	JSC-1A^22^	Apollo 14 sample 14,163	Apollo 17 sample 70,051
SiO_2_	49.10	46–49	46.20	45.7	47.3	42.20
TiO_2_	1.48	1–2	1.85	1.9	1.6	5.09
Al_2_O_3_	15.50	14.5–15.5	17.10	16.2	17.8	15.70
Fe_2_O_3_		3–4		12.4		
FeO	9.81^a^	7–7.5	11.20		10.5	12.40
MgO	8.48	8.5–9.5	6.87	8.7	9.6	10.30
CaO	10.10	10–11	9.43	10.0	11.4	11.50
Na_2_O	2.46	2.5–3	3.33	3.2	0.7	0.24
K_2_O	0.85	0.75–0.85	0.85	0.8	0.6	0.07
MnO	0.18	0.15–0.20	0.19	0.2	0.1	0.15
P_2_O_5_	0.61	0.6–0.7	0.62	0.7		
Cr_2_O_3_		0.02–0.06			0.2	

^a^Total Fe is expressed as FeO, where stated. Fe_2_O_3_ content of JSC-1A was estimated based on the material safety reference^22^*.*

JSC-1A is composed of particles of basaltic glass (49.3 area%), containing the minerals olivine (9.0 area%), plagioclase (37.1 area%), titaniferous magnetite (0.4 area%), pyroxene (< 0.1 area%), and ilmenite (< 0.1 area%)^36,37^. The particle size of JSC-1A is ≦ 1 mm^22^. The bulk density of JSC-1A used in this experiment is 1.540 g/cm^3^, exhibiting a void fraction between particles of 47% when at rest.

JSC-1A absorbs microwave energy very well compared with lunar highlands soil simulants (e.g. NU-LHT-2M) because of its dielectric properties. The dielectric properties of low loss materials and powdered samples, including lunar soil and simulants, can be measured with a cavity perturbation technique^39,40^, which is one of the most commonly used techniques under high and low temperature^41,42^. The dielectric constant (ε′) is the ability of the material to polarize, and the dielectric loss (ε′′) is the ability to transfer the microwave energy into heat in the material. Loss tangent (δ) is the ratio of dielectric loss to dielectric constant, i.e., δ = ε′′/ε′, which indicates the ability of the material to be polarized and heated^43^. Thus, a higher dielectric loss tangent allows more dielectric loss heating. The loss tangent of JSC-1A at 2.45 GHz frequency is 0.0185, while the loss tangent of lunar mare regolith (Apollo 11 and 17) is 0.0115. Since the lunar mare soil simulant has a higher dielectric loss tangent than that of lunar highlands soil simulant (e.g. NU-LHT-2M estimated to be 0.0057), it not only absorbs more microwave energy, it also heats much more rapidly. Note that the loss tangent of JSC-1A and NU-LHT-2M are estimated based on the data from Bamartz et al.^44,45^.

Given the iron content (FeO and Fe_2_O_3_ in Table 1) of lunar soil and simulants would significantly influence the dielectric properties of the material, the Apollo17 soil (12.40 wt%) and JSC-1A (11.15–12.40 wt%) should have much better microwave absorption than JSC-1 (9.81 wt%) and Apollo 14 soil (10.5 wt%), which would translate into JSC-1 and Apollo 14 soil requiring higher energy input than JSC-1A and Apollo 17 soil.

### Experiment apparatus settings and procedure

The Open University commissioned a bespoke 2.45 GHz microwave heating equipment to overcome the limitations of a conventional microwave oven. The penetration depth of microwave energy into the lunar soil is inversely proportional to the microwave frequency, i.e., higher frequency has shallow penetration depth while lower frequency has a deeper penetration depth^2^. The frequency also affects the dimension of the resonant cavity and optimal mass to be sintered/melted. As we conducted preliminary experiments using a commonly available 2.45 GHz microwave oven (i.e. a typical kitchen oven), we selected this frequency for further experiments. The equipment is capable of (i) allowing a single-mode where the microwave wavelength is focused and matched with the length of a resonant cylindrical cavity, which means the microwave energy is uniformly irradiated throughout the sample, to achieve higher energy efficiency, (ii) withstanding high temperatures, e.g. the melting temperature of lunar soil, i.e., over 1300 °C; (iii) providing a vacuumed chamber condition; (iv) controlling the input and reflective power; and (v) monitoring and recording the temperature changes of a specimen as a function of time.

Figure 1a illustrates each component of the microwave heating equipment. The magnetron generates microwave energy and sends it to the chamber through the waveguide and coaxial port. The controller allows to (i) adjust the input power from zero to 1000 W; (ii) monitor the reflected power from the chamber, the temperature of a specimen and various system feedback; (iii) pre-define the heating time; and (iv) disconnect the power if the chamber door is not sealed properly. The standard three Stub tuner and the vertical tuner allows to maximise the input power from the magnetron and adjust the hotspot position in the chamber. Volatiles in lunar soil and simulants, which could be used for producing propellant and life support (e.g. water and oxygen), can be extracted at various temperature ranges^38,46–50^. Thus, the chamber is connected to a vacuum pump capable of 10^–8^ mbar pressure with the mass spectrometer for analysing released volatiles while a specimen is being heated, although they were not used in this work.

The microwave heating experiment was carried out with the following settings and procedures.

- *Specimen material* Lunar soil simulant JSC-1A, in a powder form. The powder was not compressed; thus, the bulk density of the pristine JSC-1A powder is 1.54 g/cm^3^. This is lower than 1.65–1.66 g/cm^3^ reported by other researchers^8,45^.
- *Specimen mass* 50 g initial mass for each specimen. At least five specimens were fabricated for each input power, and the specimen that gave the highest yield was selected for the comparative analysis. The highest yield for each input power was: 100% (50 g converted from the initial 50 g mass) for 1000 W; 90% (45 g) for 800 W, 82% (41 g) for 600 W; 70% (35 g) for 400 W; and 44% (22 g) for 250 W.
- *Specimen preparation for SEM/EDS/nano-indentation and true density analysis* The thermally treated specimens were sliced vertically and then horizontally to yield 4 pieces. Subsequently, we selected one of the pieces which visually contained more melted surface. This piece was then embedded in 1-inch resin mount, polished flat to reveal the internal structure, and finally coated with a 20 nm conductive layer of carbon for further characterisation (Fig. 1c).
- *Specimen holder* A conventional-shaped alumina ceramic crucible (99.8% purity), which is transparent to microwave energy, was used. The dimensions of the crucible are 52 mm diameter on the top, 32 mm diameter on the bottom and 50 mm height. The inside of the crucible is covered with a 2 mm thick alumina ceramic paper to minimise thermal shock caused by thermal runaway and keep the powder contained even if the crucible breaks.
- *Microwave input power* Based on our previous experiments^10,14^, we have learnt that 50 g of JSC-1A simulant can be fully molten in 15 min under 1000 W input power. Thus, we have set the total input energy as 900 kJ, which translates into 900 s of heating time for 1000 W, 1125 s for 800 W, 1500 s for 600 W, 2250 s for 400 W, and 3600 s for 250 W.
- *Temperature measurement* The temperature of specimens was measured with a 5-min interval, while the extra temperature points are also measured when the 800 W and 400 W specimens heating was completed in a middle of the 5-min interval. The cylindrical chamber has two RAYTEK Miniature Infrared sensors (the temperature range of the left pyrometer is between ambient to 1000 °C protected using gallium glass, while the of the right pyrometer is between 500 and 1400 °C protected using fused quartz glass), one fused silica glass viewport and a sealed door, in the front. As a support crucible was used for positioning the specimen on the optimal hotspot position, the two pyrometers measured the side surfaces of the crucible rather than the top surface of the specimen. This means the recorded temperatures are considerably lower than the surface temperatures of the specimen.
- *The heating procedure of the specimen* A pre-defined input power is instantly fed when the power is turned on for the given time, and the tuner is manually adjusted when the reflected power increases. The waveguide and the wall of the vertical tuner are thermally controlled using pressured air (≈ 3 bar) to remove any dust inside and protect the system from the excessive heat generation. The previous experiment with computational simulation has revealed severe thermal runaway at the core of the specimen; thus, it is necessary to maintain the equipment temperature under the safe operating level. The chamber wall is water-cooled (≈ 18 °C) while the microwave power is on, so to protect the aluminium chamber from the heat. However, this setup causes higher convective and radiative heat loss with longer heating times. For example, from our numerical simulation, the predicted maximum temperature of the hotspot in each sample was quite different (e.g., > 1700 °C for 1000 W and > 1400 °C for 250 W). The temperature increase near the hotspot area was reduced from ≈ 1050 °C with 1000 W to ≈ 900 °C with 400 W and ≈ 850 °C with 250 W. Taken at face value, this could be a potential explanation as to why the 400 W and 250 W samples have much smaller glows and lower yield rates. In future experiments, a radiant barrier to the chamber could be used to improve energy efficiency by minimising the heat loss. This may produce more sintered/molten mass in less time, which would be beneficial for low input powers. The pyrometers are capable of recording the temperature continuously. However, in this experiment, the temperature was recorded every 5 min while the power is on. The pressure was kept to atmospheric values for this set of experiments.
- *The cooling procedure of the heated specimen* Once the power is turned off, the specimen is left in the chamber with natural cooling until the temperature of the crucible surface reaches 100 °C, then the chamber door is opened, and the specimen is further cooled down under ambient temperature (around 20–25 °C). Ideally, the input power during the cooling procedures should be gradually reduced to prevent any thermal shock in the microstructure of thermally treated specimen. However, in this experiment, we have set a simple power on/off to minimise the complexity of the operation.
- *Specimen analysis* The microstructures and chemical modifications of the thermally treated (sintered/molten) specimens were imaged (Fig. 1d) and analysed with a Scanning Electron Microscope (SEM) in Backscattered Electron (BSE) mode using a FEI QUANTA 200 Scanning Electron Microscope, equipped with an Oxford Instruments Energy Dispersive Spectroscopy (EDS) detector. The same specimen were subsequently measured with a CAMECA SX 100 Electron Probe Micro Analyser (EPMA) for oxide major elements quantification (Na_2_O, K_2_O, CaO, MgO, FeO, TiO_2_, Cr_2_O_3_, MnO, Al_2_O_3_, SiO_2_) of mineral phases. The entire list of analysed oxide weight % of five mineral categories for all specimens can be found as Supplementary Data 1. Working conditions were 15 kV accelerating voltage, 20 nA current, using a 10 μm defocussed beam for glass and iron-rich olivine, and a focused beam for the other mineral phases. The hardness and elastic modulus of the specimens were measured using a NANOINDENTER XP (MTS, USA). Indentations were performed using a diamond-tipped BERKOVICH indenter. The testing temperature was maintained within the range of 20–22 °C to reduce thermal drift. For each surface location tested, 64 separate indentations were performed over an area of dimensions 70 × 70 µm. Specimens were indented at a strain rate of 0.05 s^−1^ to a maximum depth of 500 nm. Elastic modulus and hardness were calculated using the Oliver-Pharr method^51^, in which a second-order polynomial is fit to the unloading section of the load–displacement data. The Poisson’s ratio of the material was assumed to be 0.3. True Density analysis of each specimen was performed by measuring the broken fragments of each specimen generated via microwave heating using the Micromeritics AccuPyc II 1340 Gas Pycnometer. The volume of each specimen was calculated using Archimedes principle^52^ 20 times for each specimen. The volume was used in conjunction with the specimen mass to calculate the specimen density.

## Supplementary Information

Supplementary Information.
